# Increased expression of costimulatory markers CD134 and CD80 on interleukin-17 producing T cells in patients with systemic lupus erythematosus

**DOI:** 10.1186/ar3100

**Published:** 2010-07-23

**Authors:** Sebastian Dolff, Daniel Quandt, Benjamin Wilde, Thorsten Feldkamp, Fan Hua, Xin Cai, Christof Specker, Andreas Kribben, Cees GM Kallenberg, Oliver Witzke

**Affiliations:** 1 Department of Nephrology, University Hospital Essen, University Duisburg-Essen, Hufelandstraße 55, 45136 Essen, Germany; 2 Department of Rheumatology and Clinical Immunology, University Medical Center Groningen, University of Groningen, Hanzeplein 1, 9700 RB Groningen, The Netherlands; 3 Department of Rheumatology and Clinical Immunology, Kliniken Essen Süd, Propsteistraße 2, 45239 Essen, Germany

## Abstract

**Introduction:**

There is growing evidence that interleukin 17 (IL-17) producing T cells are involved in the pathogenesis of systemic lupus erythematosus (SLE). Previous studies showed that increased percentages of T-cell subsets expressing the costimulatory molecules CD80 and CD134 are associated with disease activity and renal involvement in SLE. The aim of this study was to investigate the distribution and phenotypical characteristics of IL-17 producing T-cells in SLE, in particular in patients with lupus nephritis, with emphasis on the expression of CD80 and CD134.

**Methods:**

Thirty-four patients (3 male, 31 female, mean age 41 ± 15 years) fulfilling at least four of the American College of Rheumatology (ACR) revised criteria for the diagnosis of SLE and 24 healthy controls were enrolled. T-cells from the peripheral blood were analysed by fluorescence activated cell sorting (FACS) for their expression levels of CD80, CD134 and CCR6. *In vitro *stimulated CD3^+^IL17^+ ^cells were also investigated for the expression of these costimulatory markers. Finally, renal biopsies from SLE patients were evaluated for the presence of CD134 expressing T-cells.

**Results:**

Percentages of IL-17 expressing T-cells were significantly increased in patients with active disease as compared to healthy controls (1.46 ± 0.58% versus 0.93 ± 0.30%, *P *= 0.007). The percentage of IL-17 producing T-cells was correlated with disease activity as assessed by systemic lupus erythematosus disease activity index (SLEDAI) (r = 0.53, *P *= 0.003). In patients, most of the IL-17 producing T-cells were confined to the CCR6^**+ **^T-cell subset (80 ± 13%). Expression of CD80 and CD134 on the IL-17 producing T-cell subset was higher in SLE than in healthy controls (HC) (CD134: 71.78 ± 14.51% versus 51.45 ± 16.58%, *P *= 0.002; CD80: 25.5 ± 14.99% versus 14.99 ± 5.74%, *P *= 0.02). Also, patients with lupus nephritis expressed higher levels of CD134^**+ **^on CD3^+^IL-17^+ ^cells as compared to HC (72.69 ± 11.54% versus 51.45 ± 16.58%, *P *= 0.006). Furthermore, renal biopsies of lupus nephritis patients showed infiltration of CD134^+ ^T cells.

**Conclusions:**

Percentages of IL-17 expressing T-cells correlate with disease activity. Further, these cells show increased expression of costimulatory markers such as CD134 and CD80. The presence of CD134^+ ^T-cells in renal biopsies of lupus nephritis patients suggest that these cells migrate to the kidney and might contribute to inflammatory processes through IL-17 secretion.

## Introduction

Systemic lupus erythematosus (SLE) is a multiorgan autoimmune disease characterized by an imbalanced T cell homeostasis with a shift towards activated effector T-cell subsets. Two major subsets of CD4^+ ^T helper cells, Th1 and Th2, have been shown to be involved in the pathogenesis of SLE. Th1 cells secrete interferon gamma (INF-γ) and are induced by IL-12, whereas Th2 cells secrete IL-4, IL-5 and IL-13 and are induced by IL-4 [1,2].

More recently, another subset of cytokine producing T-cells, so called Th17 cells producing the cytokine IL-17, were described. IL-17 exerts its function through recruiting neutrophils and monocytes, upregulating local chemokine expression, facilitating T cell migration into tissues, and inducing immune responses [3-6]. There is increasing evidence that IL-17-producing T-cells play an important role in various autoimmune diseases including multiple sclerosis, psoriasis, rheumatoid arthritis, inflammatory bowel disease, ANCA associated vasculitis and systemic lupus erythematosus [7-11].

Recent studies demonstrated the importance of IL-17 produced by different T-cell subsets such as CD4^+ ^T-cells, CD8^+ ^T cells, CD3^+^CD4^-^CD8^- ^T-cells, and γδ T-cells in human SLE [12,13]. Furthermore, Yang *et al. *reported an association of IL17-producing T-cells and clinical features as disease activity assessed by systemic lupus erythematosus disease activity index (SLEDAI) [14]. Studies investigating the role of IL-17-producing T-cells in the pathogenesis of lupus nephritis (LN) are rare. However, studies in mice support the idea that IL-17 may contribute to renal disease, in particular lupus nephritis [14-17]. Crispin *et al. *demonstrated infiltration of IL-17^+ ^double negative T-cell in kidneys of lupus nephritis patients [12]. More recently, single-cell analysis of laser-microdissected lupus nephritis sections showed a skewing towards IL-17 [18]. Further evidence comes from urine analysis in lupus nephritis patients where IL-17 gene expression was inversely correlated with disease activity [19]. These studies demonstrated the pivotal role of IL-17 cells in the pathogenesis of lupus nephritis. In humans an overwhelming amount of IL-17 cells express the chemokine receptor CCR6 [20]. Therefore, CCR6 might be a useful phenotypic marker for the analysis of IL-17 T-cells.

Previously, we reported an association of increased levels of costimulatory markers on CD4^+ ^cells with lupus nephritis [21]. Especially, expression of CD134 was associated with disease activity and renal involvement, but a functional analysis of these CD134 expressing cells is still lacking. As a member of the tumour necrosis factor (TNF) superfamily, CD134 (OX40) provides co-stimulatory signals upon ligation to the CD134Ligand. Moreover, it is possible that CD134^+ ^T cells infiltrate kidneys and cause inflammation after ligation with CD134L which has been shown to be present on glomerular endothelial cells in SLE patients [15]. Thus, CD134 might be a pivotal surface marker to enable effector cell migration towards the kidney. The significance of CD134 for effector functions has been shown by the observation that treatment with a stimulatory anti-CD134 antibody enhances T cell expansion and differentiation to effector cells in mice [22,23]. This stimulation, apparently, promotes the secretion of IFN-γ and the upregulation of various interleukin (IL)-receptors which might lead subsequently to cytokine-mediated kidney cell damage [24]. Remarkably, increased percentages of CD134 expressing T-cells as well as Th17 cells have been found amongst effector cells in several autoimmune diseases such as Wegener's granulomatosis, rheumatoid arthritis and myasthenia gravis [7,25-28].

To elucidate the role and phenotype of IL-17 producing effector T-cells in patients with systemic lupus erythematosus, in particular with lupus nephritis, we investigated their presence and phenotypic characteristics in the present study. We analysed not only peripheral blood but also tissue from patients with lupus nephritis. We tested the hypothesis that increased expression of costimulatory molecules on these cells may promote their infiltrations into the kidney due to interaction with ligands on resident renal cells.

## Materials and methods

Thirty-four patients (3 male, 31 female, mean age 41 ± 15 years) fulfilling at least four of the American College of Rheumatology (ACR) revised criteria for the diagnosis of SLE and 24 healthy controls (mean age 46 ± 14 years) were enrolled in the study. Thirteen patients had lupus nephritis (WHO Class II: two patients, Class III: one patient, Class IV: five patients, Class V: four patients, unclassified: two patients) while 22 patients had no clinical evidence of lupus nephritis (absence of proteinuria and/or glomerular hematuria). Clinical disease activity at the time of measurement was assessed according to the systemic lupus erythematosus disease activity index (SLEDAI) (mean SLEDAI 4 ± 5). Patients with a SLEDAI ≥4 were defined as active; 19 active and 15 inactive patients were included in the study. A total of 26 patients were treated with prednisone (mean 27 ± 91 mg/d), 8 patients did not receive prednisone, and 23 patients were on a constant dose of immunomodulating drugs (azathioprin (*n *= 8), mycophenolate mofetil (*n *= 10), cyclosporin A (*n *= 2), hydroxychloroquin (*n *= 9), leflunomide (*n *= 1), rituximab (*n *= 2)), 2 patients had no medication at the time of the study. Thirteen patients had been treated with cyclophosphamide (mean dose: 2,003 ± 4,193 mg) during their disease course. Consecutive patients were included from the University Hospital of Essen and the Medical Center Essen-Süd. The study protocol was approved by the institutional review board. All patients gave informed consent for participation in this study.

### Flow cytometry

Expression level of the surface molecules on lymphocytes was assessed by four-colour surface staining. Phycoerythrin (PE), fluorenscein isothiocyanate (FITC), peridin chlorophyll protein (PerCP) and Allophycocyanin (APC)-labelled antibodies were used: CD3 (mouse IgG1, PerCP), CD4 (mouse IgG1, PerCP), CD8 (mouse IgG1 FITC), CCR6 (mouse igG1, FITC), CD134 (mouse IgG1, PE), CD80 (mouse IgG1, PE, R&D Systems, Wiesbaden, Germany) and IL-17 (mouse IgG1, APC). All antibodies were purchased from Becton Dickinson, Heidelberg, Germany, except for IL-17, which was purchased from eBioscience, San Diego, CA, USA, and CCR6 purchased from R&D Systems, Minneapolis, MN, USA. Appropriate isotype controls (Becton Dickinson) were used. Briefly, peripheral blood was stained with labelled monoclonal antibodies for 20 minutes in the dark at room temperature. The cell suspension was incubated with lysis buffer for 15 minutes and prepared as indicated. Analysis was performed with a fluorescence activated cell sorter (FACS) Calibur™ from Becton Dickinson.

### Immunostaining for intracellular cytokines

Peripheral blood mononuclear cells (PBMCs) of patients were separated by standard Ficoll-Paque density gradient centrifugation. The cells were resuspended in RPMI 1640 medium (Gibco BRL, Karlsruhe, Germany) supplemented with 10% heat inactivated fetal calf serum (Biochrom, Berlin, Germany). The cells were cultured in the absence or presence of PMA (5 ng/ml) and Ionomycin (1 μM) (Sigma-Aldrich, Seelze, Germany) for five hours. Cytokine secretion was inhibited by Brefeldin A (Ebioscience, Frankfurt, Germany). Then surface staining was performed with CD3, CD134, CD80, CCR6 and appropriate isotype controls. Cells were fixed and permeabilized by using a Cytofix/Cytoperm kit purchased from Becton Dickinson. Finally, the samples were intracellulary stained with IL-17 or an appropriate isotype control.

### Immunohistochemistry

Renal biopsies were provided by the Institute of Pathology University Hospital of Essen. Specimens were fixed in 10% neutral buffered formaline and paraffin-embedded. Sections 5 μm thick were deparaffinized in xylene and rehydrated in a series of ethanol with different concentrations (100%, 95%, 70% and 50%). Citrate buffer pH 6.0 (Zytomed, Berlin, Germany) was applied for heat induced epitope retrieval, followed by neutralization of endogenous peroxidase with 0.3% H_2_O_2_. Primary antibodies (CD3 obtained from DCS, Hamburg, Germany and CD134 obtained from Becton Dickinson) and HRP-conjugated secondary antibodies (Zytomed) were incubated on slides (each for 30 minutes) at room temperature. Washing with PBS was performed after each incubation step. A DAB substrate Kit (Zytomed) was used for visualization. Finally, the slides were slightly counterstained with hematoxylin.

### Immunofluorescence double staining

Tissues were fixed, embedded in paraffin and sectioned as indicated above. Epitope retrieval was performed with citrate buffer pH 6.0 (Zytomed). Primary antibodies against CD3 (rabbit IgG1, DCS) and CD134 (mouse IgG1, Becton Dickinson) were used and incubated for 60 minutes at room temperature simultaneously. Secondary antibodies conjugated to Cy2 and Cy3 (Dianova, Hamburg) were applied for 30 minutes. Finally, the slides were mounted with Immu Mount™ (Thermo Fisher, Kehl, Germany).

### Statistics

All values are expressed as mean ± SD. Significance for the differences between groups was determined by the Mann-Whitney U-test. Spearman's rank correlation test was applied for detecting correlations between different study parameters. A *P-*value less than 0.05 was considered significant.

## Results

### Expression of CCR6 and phenotypic features of CCR6^+ ^peripheral blood CD4^+ ^cells

In order to characterise IL-17 producing cells with a suitable marker expressed on the surface we analysed T cells for the expression of CCR6 [20]. Peripheral blood circulating CD4^+^CCR6^+ ^cells were analysed in 28 patients and 11 healthy individuals. There were no differences in the percentages of CD4^+^CCR6^+ ^cells between patients with SLE and healthy controls (19.74 ± 8.5% vs. 16.97 ± 5.6%, *P *= 0.34). Furthermore, there were no significant differences between active (*n *= 10) and inactive (*n *= 18) patients (18.63 ± 8.01% vs. 20.36 ± 9.04%, *P *= 0.46) or between patients with or without lupus nephritis (22.39 ± 8.0% vs. 17.86 ± 8.08%, *P *= 0.24). There were also no differences in of CCR6 expression between patients with and without lupus nephritis and healthy controls (22.39 ± 8.05% vs. 16.97 ± 5.67% and 17.86 ± 8.08% vs. 16.97 ± 5.67%, *P *= 0.08 and *P *= 0.76, respectively). Active and inactive patients showed no difference in levels of CCR6^**+ **^cells as compared to healthy controls (18.63 ± 8.01% vs. 16.97 ± 5.67% and 20.36 ± 9.04% vs. 16.97 ± 5.67%, *P *= 0.6 and *P *= 0.31, respectively).

The CCR6^+ ^subset was analysed for the expression of costimulatory markers CD80 and CD134. Remarkably, percentages of CD134 and CD80 expressing CCR6^**+ **^cells were significantly increased in patients with SLE in comparison to healthy controls (CD134: 67.87 ± 12.23% vs. 59.27 ± 8.18%, *P *= 0.02; CD80: 26.46 ± 12.28% vs. 13.86 ± 3.82%, *P *= 0.001).

### Expression of costimulatory markers CD134 and CD80 on CD4^+^CCR6^+ ^cells in active and inactive patients and patients with and without lupus nephritis

Patients with and without active disease, as assessed by SLEDAI, and patients with and without lupus nephritis, proven by renal biopsy, were analysed for CD80 expression on CD4^+^CCR6^+ ^cells. The percentages of CD80 expressing on CD4^+^CCR6^+ ^cells showed no difference between active and inactive patients (23.58 ± 13.41% vs. 28.06 ± 11.07%, *P *= 0.37). There was a significant difference between inactive SLE patients and healthy controls (28.06 ± 11.07% vs. 13.86 ± 3.82%, *P *= 0.0006). Expression of CD80 on CD4^+^CCR6^+ ^cells in active patients tended to be increased as compared to healthy controls (23.58 ± 13.41% vs. 13.86 ± 3.82%, *P *= 0.062). Patients with lupus nephritis showed significantly higher levels of CD80 expression on CD4^+^CCR6^+ ^cells in comparison to healthy controls (22.13 ± 10.58% vs. 18.86 ± 3.82%, *P *= 0.02). The same applied for patients without lupus nephritis (30.66 ± 12.75% vs. 18.86 ± 3.82%, *P *= 0.0009). Comparing patients with lupus nephritis and patients without lupus nephritis no difference could be observed (22.13 ± 10.58% vs. 30.66 ± 12.75%, *P *= 0.11).

Expression of CD134 on CD4^+^CCR6^+ ^cells was significantly higher in patients with inactive disease compared to healthy controls (70.89 ± 10.35% vs. 59.27 ± 8.18%, *P *= 0.0097) but not different from active patients (70.89 ± 10.35% vs. 62.14 ± 14.16%, *P *= 0.17). Patients with lupus nephritis expressed higher levels of CD134 on peripheral blood CD4^+ ^T-cells as compared to HC (68.08 ± 7.52% vs. 59.27 ± 8.18%, *P *= 0.027). Patients without lupus nephritis showed no difference in comparison to patients with lupus nephritis and healthy individuals (67.75 ± 13.46% vs. 68.08 ± 7.52%, *P *= 0.91; 67.75 ± 13.46% vs. 59.27 ± 8.18%, *P *= 0.05).

### SLE patients with active disease show increased levels of IL-17 producing T-cells in peripheral blood

**Figure 1:**
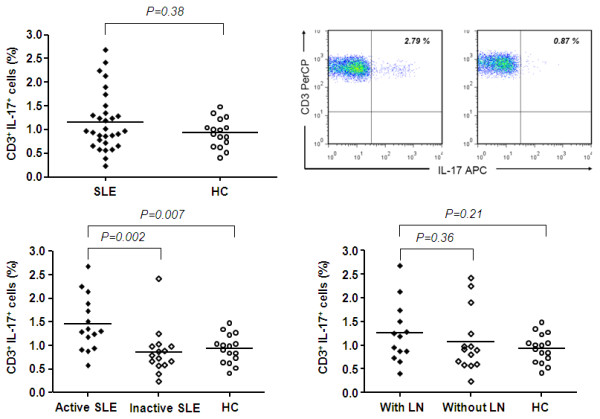
**IL-17 producing CD3^+ ^T-cells in systemic lupus erythematosus**. **(a) **Percentages of IL-17 producing CD3^+ ^cells in patients with SLE (*n *= 30) and healthy controls (HC) (*n *= 16). **(b) **A representative two colour immunofluorescence dot plot of CD3^+ ^cells showing expression levels of IL-17 from an SLE patient and a healthy control. Cells positive for both antibodies are represented in the right upper quadrant with the percentage indicated. **(c) **Percentages of IL-17 producing CD3^+ ^cells in active patients (*n *= 15), inactive patients (*n *= 15) and healthy controls (*n *= 16). **(d) **Percentages of IL-17 producing CD3^+ ^cells in patients with lupus nephritis (with LN) (*n *= 13), patients without lupus nephritis (without LN) (*n *= 14) and healthy controls (*n *= 16). Data are presented as mean value. Significance was tested by the Mann-Whitney U-test. A *P-*value less than 0.05 was considered significant.

The percentage of IL-17 producing T-cells was analysed in the peripheral blood of 30 SLE patients and 16 healthy controls. There was no significant difference between SLE patients and healthy controls (1.17 ± 0.61% vs. 0.93 ± 0.30%; *P *= 0.37). Patients with active disease had significantly elevated levels of IL-17 expressing T-cells in the peripheral blood in comparison to healthy controls (1.46 ± 0.58% vs. 0.93 ± 0.30%, *P *= 0.007). Active patients had also increased levels of IL17^+ ^T-cells as compared to inactive patients (1.46 ± 0.58% vs. 0.88 ± 0.5%, *P *= 0.002) (Figure 1). The expression of IL-17 within CD8 cells revealed an expression of 0.9 ± 0.5% (*n *= 5).

### *Ex vivo *IL-17 production of CD3^+ ^cells correlates with disease activity and is independent of renal involvement

**Figure 2:**
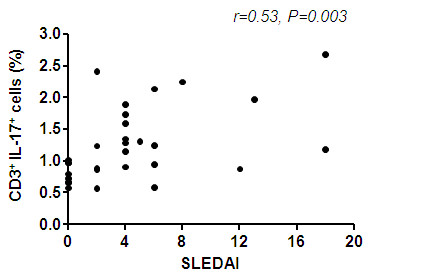
**Correlation of IL-17 producing CD3^+ ^T-cells and disease activity assessed by SLEDAI**. Correlation between percentages of IL-17 producing T cells for all samples taken (*n *= 30) and disease activity as assessed by the Systemic Lupus Erythematosus Disease Activity Index (SLEDAI). Spearman analysis was performed to calculate the correlation. A *P-*value less than 0.05 was considered significant.

The percentage of IL-17 producing CD3^+ ^cells correlated significantly with disease activity (*P *= 0.003, r = 0.53) (Figure 2). To study the influence of medication we compared the proportion of IL-17 expressing cells in patients on prednisone alone versus patients on a combination of immunosuppressants. There was no difference between these groups. This observation could be confirmed in a follow-up in 12 patients. Changes over time (21 ± 13 weeks) of IL-17 expression were associated with changes in disease activity (r = 0.81, *P *= 0.001). Expression of IL-17 in patients with lupus nephritis (*n *= 13) was not different as compared to patients without lupus nephritis (*n *= 14) (1.29 ± 0.65% vs. 1.08 ± 0.57%, *P *= 0.34). A subanalysis revealed also no difference regarding the the expression of IL-17 between patients with a class IV LN and patients with class V (0.94 ± 0.53% vs. 1.62 ± 0.94%, *P *= 0.25). There was also no association with other histological features. Further, there was no correlation between expression of IL-17 and anti-dsDNA titres or complement levels. Patients with lupus nephritis showed no difference to healthy controls (0.93 ± 0.30%, *P *= 0.15).

### Phenotypic features of IL-17^+ ^T-cells in patients with SLE

**Figure 3:**
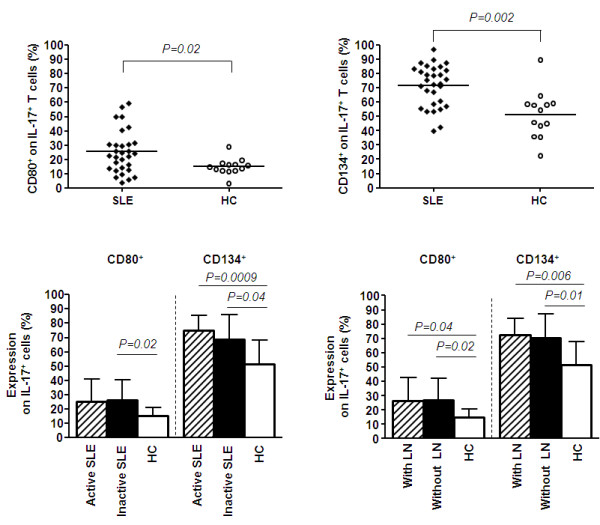
**Expression of costimulatory markers CD80 and CD134 on IL-17^+ ^CD3^+ ^cells in SLE**. **(a) **Percentages of CD80^+ ^IL-17 producing CD3^+ ^cells in patients with SLE (*n *= 30) and healthy controls (HC) (*n *= 13). **(b) **The expression of CD134^+ ^on IL-17 producing CD3^+ ^cells from patients with SLE (*n *= 30) and healthy controls (HC) (*n *= 13). **(c) **Percentages of CD80^+ ^and CD 134^+ ^IL-17 producing T cells form active (*n *= 15) and inactive (*n *= 15) patients with SLE and healthy controls (*n *= 13). **(d) **Expression of CD80^+ ^and CD 134^+ ^on IL-17 producing T cells from patients with lupus nephritis (with LN) (*n *= 13), patients without lupus nephritis (without LN) (*n *= 14) and healthy controls (*n *= 13). Data are shown as mean value. Significance was tested by the Mann-Whitney U-test. A *P-*value less than 0.05 was considered significant.

The expression of costimulatory markers CD80 and CD134 was analysed within the IL-17^+ ^subset in patients and healthy controls. The percentages of CD134 and CD80 expressing IL-17 producing T-cells were significantly increased in SLE patients in comparison to healthy controls (CD134: 71.78 ± 14.51% vs. 51.45 ± 16.58%, *P *= 0.002; CD80: 25.5 ± 14.99% vs. 14.99 ± 5.74%, *P *= 0.02) (Figure 3a, b). A subanalysis in SLE patients (*n *= 5) showed that only 1.7 ± 1.8% IL-17 producing T-cells were double positive for CD80 and CD134. There was no significant correlation between the proportion of CD134 and CD80 and IL-17 expression (*P *= 0.8 and *P *= 0.6, respectively).

### Expression of costimulatory markers CD134 and CD80 on IL17-producing T-cells in active and inactive patients and patients with and without lupus nephritis

Patients with and without active disease were analysed for CD80 expression on IL-17 producing T-cells. The percentages of CD80 expressing IL-17 producing T cells showed no differences between active and inactive patients (25.02 ± 16.01% vs. 25.97 ± 14.46%, *P *= 0.87). There was a significant difference between inactive SLE patients and healthy controls (25.97 ± 14.46% vs. 14.99 ± 5.74%, *P *= 0.02). Expression of CD80 on IL-17 producing T cells in active patients tended to be increased as compared to healthy controls (25.02 ± 16.01% vs. 14.99 ± 5.74%, *P *= 0.07). Patients were further analysed based on the presence of lupus nephritis. Patients with and without lupus nephritis showed significantly higher levels of CD80 expression on IL17^+ ^cells as compared to healthy controls (26.71 ± 15.85% and 26.90 ± 15.06% vs. 14.99 ± 5.74%, *P *= 0.04 and *P *= 0.02, respectively).There was no difference in the expression of CD80 on IL17^+ ^cells between patients with and without lupus nephritis (26.71 ± 15.85% vs. 26.90 ± 15.06%, *P *= 0.85) (Figure 3c and 3d). No significant difference could be observed comparing the expression of CD80 on IL-17 producing T-cells in patients with class IV and V lupus nephritis (36.25 ± 18.66% vs. 21.93 ± 9.18%, *P *= 0.63). Interestingly, there was a correlation between CD80 expression on IL-17 producing T-cells and anti-dsDNA titres and decreased C3 levels, respectively (r = 0.6, *P *= 0.0003 and r = -0.5, *P *= 0.01).

Expression of CD134 on IL-17 producing T cells was significantly higher in patients with active disease as compared to healthy controls (74.87 ± 10.64% vs. 51.54 ± 16.58%, *P *= 0.0009). Also in patients with inactive disease expression of CD134 on IL-17 producing T-cells was higher as compared to healthy controls (68.69 ± 17.39% vs. 51.45 ± 16.58%, *P *= 0.0382), but it did not differ from active patients (68.69 ± 17.39% vs. 74.87 ± 10.64%, *P *= 0.34). Patients with lupus nephritis expressed higher levels of CD134 on peripheral T cells as compared to healthy controls (72.69 ± 11.54% vs. 51.45 ± 16.58%, *P *= 0.0056). Patients without lupus nephritis also showed higher levels of CD134 on IL-17 producing T cells in comparison to healthy individuals (70.27 ± 17.18% vs. 51.45 ± 16.58%, *P *= 0.01). No difference could be observed between patients with and without lupus nephritis (72.69 ± 11.54% vs.70.27 ± 17.18%, *P *= 0.91) (Figure 3d). Further, expression of CD134 on IL-17 producing T-cells showed no significant differences between class IV and V lupus nephritis (69.05 ± 10.97% vs. 78.27 ± 6.51%, *P *= 0.23).

### Immunohistochemistry (IHC) staining for CD134 in inflamed tissue

**Figure 4:**
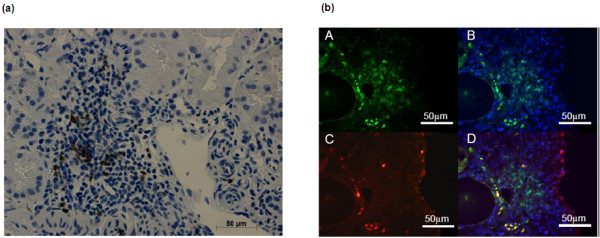
**Kidney CD134^+ ^CD3^+ ^infiltrating T-cells in lupus nephritis**. **(a) **Representative renal biopsy of an SLE patient with lupus nephritis (WHO class IV). The biopsy was stained for CD134 using immunohistochemistry. **(b) **Staining of the biopsy for CD3^+ ^and CD134^+ ^cells by immunofluorescence. CD3 (A), CD3/DAPI (B), CD134 (C) and colocalization of CD134 with CD3 (D).

To determine whether CD134^+ ^T-cells were present in inflammed organs of SLE patients, four renal biopsies were stained for CD134 and CD3. CD134^+ ^cells were located within or in the neighbourhood of small vessels and tubulointerstitial lymphocyte infiltrates. Single cells were found around glomeruli. Serial sections revealed a colocalization with CD3^+ ^T-cells; this could be confirmed by immunofluorescent double staining with CD3 and CD134 (Figure 4).

## Discussion

The results of this study suggest that IL-17 producing cells play a pivotal role in the pathogenesis of systemic lupus erythematosus. *In vitro *stimulated CD3^+ ^cells from active SLE patients produced significantly higher levels of IL-17 as compared to healthy controls. Remarkably, only T cells isolated from active patients produced higher levels of IL-17 than controls. The association between IL-17 production and disease activity was further supported by a significant correlation of IL-17 expression and disease activity as assessed by SLEDAI. These findings are in accordance with a large previous study by Yang *et al. *in which 50 patients were enrolled [14]. In addition, Wong *et al. *demonstrated increased circulating plasma concentrations of IL-17 in SLE patients as compared to healthy controls. In contrast to our study a correlation of plasma levels of IL-17 and disease activity could only be found in patients without renal disease [29]. Both studies lack a detailed subanalysis of patients with and without lupus nephritis. Therefore, a subanalysis was performed on the presence of lupus nephritis in this study. Patients with biopsy proven lupus nephritis were compared to patients without renal involvement. However, a significant difference in the amount of IL17-producing peripheral CD3^+ ^cells could not be found between these groups which could be related to the rather small size and heterogeneous composition of this group of biopsy proven lupus nephritis patients. Moreover, the impact of immunosuppressive drugs remains uncertain although we found no difference in patients on prednisone versus patients with a combination of immunosuppressive drugs regarding either IL-17 expression or expression of costimulatory molecules (data not shown). There is growing evidence in murine models that IL-17 plays a crucial role in the pathogenesis of renal diseases such as lupus nephritis [16,17]. These results can be explained by the hypothetical migration of IL-17 cells into inflamed kidneys.

The present study reveals that IL-17 cells express significantly higher amounts of the costimulatory markers CD80 and CD134. *Ex vivo *stimulation has been shown to upregulate the expression of CD134 over time but the significant difference between active patients and controls seem to be associated with disease activity according to our findings [30]. In addition, we detected CD134^+ ^T-cells in lupus nephritis biopsies. These findings have been confirmed by Zhou *et al. *in a larger analysis 40 kidney biopsies [31]. Possibly, in humans these CD134^+ ^T-cells infiltrate the kidney after ligation with CD134L expressed by endothelial cells which could subsequently lead to IL-17 mediated renal injury. Blocking CD134/CD134Ligand interaction as a therapeutic intervention has been successfully used in lupus mice [30]. However, the role of IL-17 has not been investigated in that study. Interestingly, a recent report investigating the influence of CD134/CD134Ligand interaction on IL-17 cytokine production suggests that IL-17 production is downregulated after ligation of CD134 [32]. This could be interpreted as a negative feedback loop for the effector function of CD134^+ ^cells but the detailed mechanisms remain unclear. An important regulatory role of Th17 cells through the CD28/CD80 pathway was also discussed in a murine model [33].

Taken together we demonstrated that IL-17 producing cells are closely linked to disease activity in SLE patients and express high levels of the costimulatory markers CD80 and CD134. These new subsets of IL-17 cells might be important in human lupus nephritis.

## Conclusions

The presence of CD134^+ ^T-cells in renal biopsies of lupus nephritis patients suggest that these cells migrate to the kidney and might contribute to inflammatory processes through IL-17 secretion. Further studies are necessary to dissect pathogenic role of IL-17 in lupus in order to establish IL-17 as a therapeutic target in SLE.

## Abbreviations

ACR: American College of Rheumatology; APC: Allophycocyanin; DAPI: 4',6-diamidino-2-phenylindol; FACS: fluorescence activated cell sorting; FITC: fluorescein isothiocyanate; HC: health controls; IL-17: Interleukin 17; LN: lupus nephritis; PBS: phosphate buffered saline; PE: phycoerythrin; PerCP: peridin chlorophyll protein; PBMC: peripheral blood mononuclear cell; SLE: systemic lupus erythematosus; SLEDAI: systemic lupus erythematosus disease activity index.

## Competing interests

The authors declare that they have no competing interests.

## Authors' contributions

All authors contributed to the design, acquisition and interpretation of data. DQ performed the statistical analysis. SD, CK and OW drafted the manuscript. DQ, FH, XC and BW carried out flowcytometry and immunohistochemistry experiments. TF, CS and AK assessed and participated in the interpretation of the clinical data. All authors read and approved the final manuscript.

## References

[B1] Abbas AK Murphy KM Sher A Functional diversity of helper T lymphocytes Nature 1996 383 787 793 10.1038/383787a0 8893001

[B2] Viallard JF Pellegrin JL Ranchin V Schaeverbeke T Dehais J Longy-Boursier M Ragnaud JM Leng B Moreau JF Th1 (IL-2, interferon-gamma (IFN-gamma)) and Th2 (IL-10, IL-4) cytokine production by peripheral blood mononuclear cells (PBMC) from patients with systemic lupus erythematosus (SLE) Clin Exp Immunol 1999 115 189 195 10.1046/j.1365-2249.1999.00766.x 9933441 PMC1905189

[B3] Annunziato F Cosmi L Santarlasci V Maggi L Liotta F Mazzinghi B Parente E Fili L Ferri S Frosali F Giudici F Romagnani P Parronchi P Tonelli F Maggi E Romagnani S Phenotypic and functional features of human Th17 cells J Exp Med 2007 204 1849 1861 10.1084/jem.20070663 17635957 PMC2118657

[B4] Laan M Cui ZH Hoshino H Lotvall J Sjostrand M Gruenert DC Skoogh BE Linden A Neutrophil recruitment by human IL-17 via C-X-C chemokine release in the airways J Immunol 1999 162 2347 2352 9973514

[B5] Witowski J Pawlaczyk K Breborowicz A Scheuren A Kuzlan-Pawlaczyk M Wisniewska J Polubinska A Friess H Gahl GM Frei U Jörres A IL-17 stimulates intraperitoneal neutrophil infiltration through the release of GRO alpha chemokine from mesothelial cells J Immunol 2000 165 5814 5821 11067941 10.4049/jimmunol.165.10.5814

[B6] Woltman AM de Haij S Boonstra JG Gobin SJ Daha MR van Kooten C Interleukin-17 and CD40-ligand synergistically enhance cytokine and chemokine production by renal epithelial cells J Am Soc Nephrol 2000 11 2044 2055 11053480 10.1681/ASN.V11112044

[B7] Abdulahad WH Stegeman CA Limburg PC Kallenberg CG Skewed distribution of Th17 lymphocytes in patients with Wegener's granulomatosis in remission Arthritis Rheum 2008 58 2196 2205 10.1002/art.23557 18576340

[B8] Fitch E Harper E Skorcheva I Kurtz SE Blauvelt A Pathophysiology of psoriasis: recent advances on IL-23 and Th17 cytokines Curr Rheumatol Rep 2007 9 461 467 10.1007/s11926-007-0075-1 18177599 PMC2893221

[B9] Kebir H Kreymborg K Ifergan I Dodelet-Devillers A Cayrol R Bernard M Giuliani F Arbour N Becher B Prat A Human TH17 lymphocytes promote blood-brain barrier disruption and central nervous system inflammation Nat Med 2007 13 1173 1175 10.1038/nm1651 17828272 PMC5114125

[B10] Schmechel S Konrad A Diegelmann J Glas J Wetzke M Paschos E Lohse P Goke B Brand S Linking genetic susceptibility to Crohn's disease with Th17 cell function: IL-22 serum levels are increased in Crohn's disease and correlate with disease activity and IL23R genotype status Inflamm Bowel Dis 2008 14 204 212 10.1002/ibd.20315 18022867

[B11] Shen H Goodall JC Hill Gaston JS Frequency and phenotype of peripheral blood Th17 cells in ankylosing spondylitis and rheumatoid arthritis Arthritis Rheum 2009 60 1647 1656 10.1002/art.24568 19479869

[B12] Crispin JC Oukka M Bayliss G Cohen RA Van Beek CA Stillman IE Kyttaris VC Juang YT Tsokos GC Expanded double negative T cells in patients with systemic lupus erythematosus produce IL-17 and infiltrate the kidneys J Immunol 2008 181 8761 8766 19050297 10.4049/jimmunol.181.12.8761 PMC2596652

[B13] Crispin JC Tsokos GC Human TCR-alpha beta+ CD4- CD8- T cells can derive from CD8+ T cells and display an inflammatory effector phenotype J Immunol 2009 183 4675 4681 10.4049/jimmunol.0901533 19734235 PMC2878279

[B14] Yang J Chu Y Yang X Gao D Zhu L Yang X Wan L Li M Th17 and natural Treg cell population dynamics in systemic lupus erythematosus Arthritis Rheum 2009 60 1472 1483 10.1002/art.24499 19404966

[B15] Aten J Roos A Claessen N Schilder-Tol EJ Ten Berge IJ Weening JJ Strong and selective glomerular localization of CD134 ligand and TNF receptor-1 in proliferative lupus nephritis J Am Soc Nephrol 2000 11 1426 1438 10906156 10.1681/ASN.V1181426

[B16] Paust HJ Turner JE Steinmetz OM Peters A Heymann F Holscher C Wolf G Kurts C Mittrucker HW Stahl RA Panzer U The IL-23/Th17 axis contributes to renal injury in experimental glomerulonephritis J Am Soc Nephrol 2009 20 969 979 10.1681/ASN.2008050556 19339380 PMC2678032

[B17] Zhang Z Kyttaris VC Tsokos GC The role of IL-23/IL-17 axis in lupus nephritis J Immunol 2009 183 3160 3169 10.4049/jimmunol.0900385 19657089 PMC2766304

[B18] Wang Y Ito S Chino Y Goto D Matsumoto I Murata H Tsutsumi A Hayashi T Uchida K Usui J Yamagata K Sumida T Laser microdissection-based analysis of cytokine balance in the kidneys of patients with lupus nephritis Clin Exp Immunol 2010 159 1 10 10.1111/j.1365-2249.2009.04031.x 19807734 PMC2802690

[B19] Kwan BC Tam LS Lai KB Lai FM Li EK Wang G Chow KM Li PK Szeto CC The gene expression of type 17 T-helper cell-related cytokines in the urinary sediment of patients with systemic lupus erythematosus Rheumatology (Oxford) 2009 48 1491 1497 10.1093/rheumatology/kep255 19773408

[B20] Singh SP Zhang HH Foley JF Hedrick MN Farber JM Human T cells that are able to produce IL-17 express the chemokine receptor CCR6 J Immunol 2008 180 214 221 18097022 10.4049/jimmunol.180.1.214

[B21] Patschan S Dolff S Kribben A Durig J Patschan D Wilde B Specker C Philipp T Witzke O CD134 expression on CD4+ T cells is associated with nephritis and disease activity in patients with systemic lupus erythematosus Clin Exp Immunol 2006 145 235 242 10.1111/j.1365-2249.2006.03141.x 16879242 PMC1809690

[B22] Lathrop SK Huddleston CA Dullforce PA Montfort MJ Weinberg AD Parker DC A signal through OX40 (CD134) allows anergic, autoreactive T cells to acquire effector cell functions J Immunol 2004 172 6735 6743 15153490 10.4049/jimmunol.172.11.6735

[B23] Huddleston CA Weinberg AD Parker DC OX40 (CD134) engagement drives differentiation of CD4+ T cells to effector cells Eur J Immunol 2006 36 1093 1103 10.1002/eji.200535637 16541471

[B24] Williams CA Murray SE Weinberg AD Parker DC OX40-mediated differentiation to effector function requires IL-2 receptor signaling but not CD28, CD40, IL-12Rbeta2, or T-bet J Immunol 2007 178 7694 7702 17548606 10.4049/jimmunol.178.12.7694

[B25] Wilde B Dolff S Cai X Specker C Becker J Totsch M Costabel U Durig J Kribben A Tervaert JW Schmid KW Witzke O CD4+CD25+ T-cell populations expressing CD134 and GITR are associated with disease activity in patients with Wegener's granulomatosis Nephrol Dial Transplant 2009 24 161 171 10.1093/ndt/gfn461 18723571

[B26] Xiaoyan Z Pirskanen R Malmstrom V Lefvert AK Expression of OX40 (CD134) on CD4+ T-cells from patients with myasthenia gravis Clin Exp Immunol 2006 143 110 116 10.1111/j.1365-2249.2005.02955.x 16367941 PMC1809569

[B27] Giacomelli R Passacantando A Perricone R Parzanese I Rascente M Minisola G Tonietti G T lymphocytes in the synovial fluid of patients with active rheumatoid arthritis display CD134-OX40 surface antigen Clin Exp Rheumatol 2001 19 317 320 11407087

[B28] Chabaud M Durand JM Buchs N Fossiez F Page G Frappart L Miossec P Human interleukin-17: A T cell-derived proinflammatory cytokine produced by the rheumatoid synovium Arthritis Rheum 1999 42 963 970 10.1002/1529-0131(199905)42:5<963::AID-ANR15>3.0.CO;2-E 10323452

[B29] Wong CK Lit LC Tam LS Li EK Wong PT Lam CW Hyperproduction of IL-23 and IL-17 in patients with systemic lupus erythematosus: implications for Th17-mediated inflammation in auto-immunity Clin Immunol 2008 127 385 393 10.1016/j.clim.2008.01.019 18373953

[B30] Zhou YB Ye RG Li YJ Xie CM Targeting the CD134-CD134L interaction using anti-CD134 and/or rhCD134 fusion protein as a possible strategy to prevent lupus nephritis Rheumatol Int 2009 29 417 425 10.1007/s00296-008-0697-2 18802705

[B31] Zhou Y Wu Y Ye R Li Y Xie C Expression and role of CD134 and NF-KB in renal tissue of lupus nephritis Ann Rheum Dis 2007 66 Suppl II 318 (abstract)

[B32] Li J Li L Shang X Benson J Merle Elloso M Schantz A Bracht M Orlovsky Y Sweet R Negative regulation of IL-17 production by OX40/OX40L interaction Cell Immunol 2008 253 31 37 10.1016/j.cellimm.2008.04.010 18501882

[B33] Bouguermouh S Fortin G Baba N Rubio M Sarfati M CD28 co-stimulation down regulates Th17 development PLoS One 2009 4 e5087 10.1371/journal.pone.0005087 19333372 PMC2658739

